# Oncologists' attitudes toward KRAS testing: a multisite study

**DOI:** 10.1002/cam4.135

**Published:** 2013-10-02

**Authors:** Julie N Harris, Petra Liljestrand, Gwen L Alexander, Katrina A B Goddard, Tia Kauffman, Tatjana Kolevska, Catherine McCarty, Suzanne O'Neill, Pamala Pawloski, Alanna Rahm, Andrew Williams, Carol P Somkin

**Affiliations:** 1 Division of Research, Kaiser Permanente Northern CaliforniaOakland, California; 2 Department of Public Health Sciences, Henry Ford Health SystemDetroit, Michigan; 3 Center for Health Research, Kaiser Permanente NorthwestPortland, Oregon; 4 Kaiser Permanente Medical CenterVallejo, California; 5 Marshfield Clinic Research FoundationMarshfield, Wisconsin; 6 Essentia Institute of Rural HealthDuluth, Minnesota; 7 Lombardi Comprehensive Cancer CenterWashington, District of Columbia; 8 HealthPartners Research FoundationMinneapolis, Minnesota; 9 Institute for Health Research, Kaiser Permanente ColoradoDenver, Colorado; 10 Center for Health Research, Kaiser Permanente HawaiiHonolulu, Hawaii

**Keywords:** Cancer genetics, colorectal cancer, psychosocial studies

## Abstract

Recent discoveries promise increasingly to help oncologists individually tailor anticancer therapy to their patients’ molecular tumor characteristics. One such promising molecular diagnostic is Kirsten ras (KRAS) tumor mutation testing for metastatic colorectal cancer (mCRC) patients. In the current study, we examined how and why physicians adopt KRAS testing and how they subsequently utilize the information when discussing treatment strategies with patients. We conducted 34 semi-structured in-person or telephone interviews with oncologists from seven different health plans. Each interview was audiotaped, transcribed, and coded using qualitative research methods. Information and salient themes relating to the research questions were summarized for each interview. All of the oncologists in this study reported using the KRAS test at the time of the interview. Most appeared to have adopted the test rapidly, within 6 months of the publication of National Clinical Guidelines. Oncologists chose to administer the test at various time points, although the majority ordered the test at the time their patient was diagnosed with mCRC. While oncologists expressed a range of opinions about the KRAS test, there was a general consensus that the test was useful and provided benefits to mCRC patients. The rapid adoption and enthusiasm for KRAS suggests that these types of tests may be filling an important informational need for oncologists when making treatment decisions. Future research should focus on the informational needs of patients around this test and whether patients feel informed or confident with their physicians’ use of these tests to determine treatment access.

## Introduction

Oncologists and late-stage cancer patients face a complex set of treatment decisions. Recent discoveries promise increasingly to help oncologists individually tailor anticancer therapy to their patients’ molecular tumor characteristics. One such promising molecular diagnostic is Kirsten ras (KRAS) tumor mutation testing for metastatic colorectal cancer (mCRC) patients. Retrospective analyses and subsequent data from clinical trials [1–5] show that individuals with mutations in the KRAS gene do not respond to certain types of treatment. Specifically, such patients do not respond to a class of biologics targeting anti-epidermal growth factor receptor (EGFR) antibody therapy. Approximately 40% of mCRC patients are likely to have a KRAS mutation in their tumor [6, 7], suggesting treatment should be tailored based on KRAS status. As a result of this data, the National Comprehensive Cancer Network (NCCN) [8] and the American Society of Clinical Oncologists (ASCO) [9] released statements in 2008 and 2009, respectively, recommending the use of KRAS testing prior to administration of anti-EGFR therapies [9]. Additionally, the Food and Drug Administration added KRAS information to anti-EGFR labels in 2009 [10].

While there have been two recent studies documenting the rapid adoption of KRAS testing domestically and internationally [11, 12], we still know very little about how oncologists use and communicate about this test in clinical practice. In the current study, we examined how and why physicians adopt KRAS testing and how they subsequently utilize the information when discussing treatment strategies with patients. The complexity of the risk information contained within the test may be difficult for doctors to interpret and patients to comprehend, thus impacting patients’ ability to share in their treatment decisions [13, 14]. Furthermore, while clinical guidelines exist, there are still questions about the timing of KRAS test administration [15, 16]. The NCCN and ASCO guidelines are somewhat divergent on this point with the ASCO guidelines stating that “all patients with metastatic colorectal carcinoma who are candidates for anti-EGFR antibody therapy should have their tumor tested for KRAS mutations…” [9]. In contrast the NCCN guidelines state that “…the panel strongly recommends genotyping of tumor tissue…in all patients with metastatic colorectal cancer *at the time of diagnosis of stage IV disease* (NCCN Guidelines Version 1.2011, p. MS-3)” [17]. A recent study, using KRAS utilization data from 2004 to 2009, suggests that the time interval between mCRC diagnosis and administration of the KRAS test has decreased from 36 months to 9 months [12]. It is important to understand these issues in order to develop better clinical guidelines, educational programs, and procedures to assist patients and physicians in communicating about KRAS testing and other emerging molecular diagnostics in oncology practice.

This study is part of a larger multisite study called the Comparative Effectiveness Research in Genomics and Personalized Medicine for Colon Cancer (CERGEN) study examining multiple aspects of colorectal cancer genomic medicine. The objectives of the current study were to examine oncologists’ (a) reasons for (or against) KRAS test adoption; (b) current use of KRAS testing; (c) perceived test benefits and concerns; (d) communication to patients about the test; and (e) understanding of clinical guidelines.

## Methods

The CERGEN study is a multidisciplinary comparative effectiveness research study that innovatively combines evidence generation with evidence synthesis in the context of cancer genomic medicine. The CERGEN study team includes investigators from seven participating Cancer Research Network (CRN) sites [18] and collaborative partners from academic institutions. Data collection occurred at the seven CRN sites: Kaiser Permanente Northwest (Portland and Washington) (KPNW), Kaiser Permanente Northern California (KPNC), Kaiser Permanente Colorado (KPCO), Kaiser Permanente Hawaii (KPHI), Henry Ford Health System (Michigan) (HFHS), Marshfield Clinic Research Foundation (Wisconsin) (MCRF), and Health Partners Research Foundation (Minnesota) (HPRF). All sites are also members of the HMO Research Network (http://www.hmoresearchnetwork.org) and are integrated healthcare systems, providing comprehensive medical care to a defined population of more than six million people.

This study was approved by the Institutional Review Boards (IRB) at KPNW, KPHI, KPCO, MCRF, and HFHS. The IRBs for the remaining sites ceded authority to the KPNW IRB.

### Study design

We conducted semi-structured in-person or telephone interviews with oncologists from each of the seven different health plans. A purposive sampling technique was used to identify oncologists with practices serving mCRC patients from each of the seven sites. Key oncology leaders in each of the seven systems were identified and they were asked to provide contact information for potential oncologists to participate in the study. All oncologists interviewed practiced in one of the seven integrated healthcare systems participating in the study. Each interview lasted ∼20 min (range: 7–46 min), addressing current KRAS test utilization, costs, barriers/facilitators to test adoption, doctor–patient communication related to the KRAS test, and presence and adherence to the institutional test guidelines or policy. All physician interviews were conducted between March and December 2010.

Interviews were conducted by the first two authors. Each interview was audiotaped, transcribed, and entered into the Atlas.ti software, version 6.2 [19] for purposes of coding, sorting, and retrieving data for analysis. The interview guide was modified modestly over the course of the study in response to emerging themes. Information and salient themes relating to the research questions were summarized for each interview. The data were analyzed inductively, within the framework of the constant comparative method [20–23] – a standard qualitative analytic technique in which theory is generated from data.

Analysis of physician data focused on responses addressing five research questions:

1. To what extent have oncologists adopted the KRAS test?
2. At what point in the clinical process do they test for KRAS?
3. What are the perceived barriers and facilitators to test use?
4. How do oncologists incorporate patients’ perspectives into the treatment decision-making process?
5. To what extent are oncologists adhering to guidelines for KRAS testing?

## Results

### Sample characteristics

Of 54 medical oncologists contacted, 20 either declined or did not respond to our contact. We interviewed three oncologists from KPHI, 5 from HFHS, 4 from each of four sites (HPRF, KPNW, KPCO, MCRF), and 10 from KPNC, for a total of 34 interviewed. More physicians were interviewed from KPNC as there was a larger pool of oncologists. Eight oncologists were women and 26 were men, and all treated other types of cancer in addition to CRC. Their length of practice since completion of residency spanned a broad range from 2 to 33 years. The majority had extensive clinical trials experience.

### Test adoption

#### Extent

All of the oncologists in this study reported using the KRAS test at the time of the interview. Most appeared to have adopted the test rapidly, within 6 months of the publication of the ASCO provisional clinical opinion [9], or the ASCO conference at which the opinion was initially discussed. Some physicians reported that they began using KRAS testing with their patients either after the initial publications regarding KRAS efficacy were released or they referenced reports of the KRAS test clinical trial results which preceded the ASCO opinion [3, 24, 25].

#### Who gets tested?

One salient dimension of the adoption process related to how physicians determine which of the patients to test. Nearly all of the oncologists reported that they tested all mCRC patients, with a few caveats. Most physicians stated that they would not test patients who decline chemotherapy, whose performance status make them poor candidates for anti-EGFR therapy, or who are at risk for complications (e.g., recent surgery, serious comorbidity, advanced age). Less frequently cited reasons for excluding patients from testing were insurance coverage and lack of tissue. Only one physician tested every mCRC patient, regardless of overall health.

#### Timing of test

**Figure 1:**
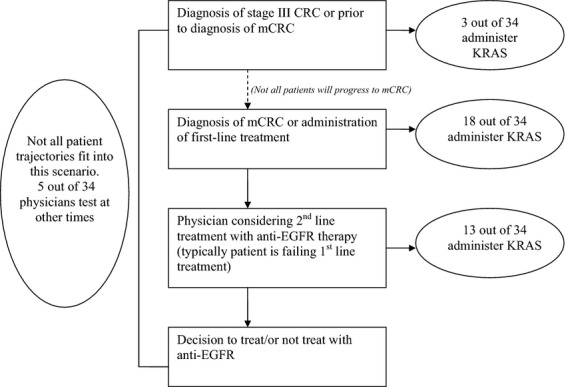
Physicians’ description of the timing of Kirsten ras (KRAS) test administration in their clinical practice. Numbers do not sum to 34 because some physicians provided more than one answer as to when they administer the KRAS test.

As shown in Figure 1, physicians chose to administer the KRAS test at various time points, although most physicians requested the test at the time their patient was diagnosed with mCRC. More than half (*n* = 18) indicated that they ordered the KRAS test when the cancer became metastatic or during first-line treatment for metastatic disease (Fig. 1). Testing “upfront,” as they explained, allowed the oncologist to articulate a relevant and personalized treatment plan and, for some, to discuss the plan with the patient in advance.

Physicians also indicated that testing upfront saves time and difficulty in trying to locate a biopsy or pathology report that might be a few years old, which can delay treatment. Finally, several physicians indicated that some of their patients actually requested the test early in the treatment process. As previously mentioned, some physicians explained that they tested patients at various different times in the process. Each of the time points that they noted testing was reported in Figure 1, so responses do not sum to 34.

About 40% (*n* = 13) of physicians indicated that they usually ordered the test after the patient had undergone conventional treatment and anti-EGFR treatment was being considered (Fig. 1). The main reason given for that timing was cost-effectiveness.

A few physicians said that they tested patients prior to developing mCRC. One physician reported occasionally testing patients without evidence of metastases, when they are at high risk for developing metastatic disease (44) or if they request the test. Similarly, another physician explained that she or he might test at stage III (or even stage II) since 30% of these (stage III) patients are likely to progress to stage IV, and it may be difficult to retrieve tumor blocks several years later.

Finally, five physicians described patient trajectories that do not easily fit into one of these patterns, emphasizing the physicians’ reliance on his or her medical judgment. In some instances a patient was being considered for anti-EGFR treatment but there was no prior biopsy or pathology report. At that point “… they usually don't re-biopsy them just to get a piece of sample for KRAS testing. Because clinically, the patient doesn't have much [time] to live … it's kind of hard to justify, you know, doing a biopsy in that condition. So a lot of them, I'll treat empirically” (33).

### Benefits of KRAS testing

All oncologists endorsed the value of the KRAS test for their patients. They typically viewed the test as standard of care and a fairly risk-free endeavor when used on existing biopsies, and noted several important benefits of the test, along with representative quotes explaining each benefit (Table 1). A key benefit mentioned was the ability to identify patients who are mutation positive, making them unlikely to benefit from anti-EGFR therapies. They also mentioned the advantage of reducing or eliminating toxicities from ineffective treatments, and conserving the expense of treating patients with a very costly drug that would likely not be beneficial. A few physicians noted that the test could serve as an agent of hope for patients who are mutation negative as it provided them with additional treatment options. Several also commented that the test helped support their development of treatment plans and facilitated doctor–patient communication.

**Table 1:** Perceived benefits of the KRAS test

Benefits of KRAS testing	Explanation/quote
Helps develop treatment plan	“This is one of the best tests … it's straightforward. It tells you yes or no, right? It's not ambiguous” (23).
Facilitates doctor–patient communication	“You can have an educated discussion and say, Hey, look, this is your mutation status and you will or you will not benefit from anti-EGFR-directed monoclonal treatment” (77).
Eliminates toxicities of ineffective treatment	“We use it in patients who are metastatic, who the whole goal of therapy is palliative. And for them to get these many side effects with no benefit doesn't make sense” (14).
Conserves resources	“For us … cost is not an issue. But if are really on the outside, you are wasting money, you are really putting – you are just wasting money down the drain so to speak if you did not do the test and gave the patient cetuximab. But in effect, you are really wasting national resources” (17).
Source of hope for patients	Patients have mostly a “very, very favorable reaction [to the test]. You know, they say, Well, you know, hopefully it will work. Hopefully it will be the wild-type and it'll work.” I do explain all that to them. I do explain we're going to look for wild-type and mutant, you know” (20).

### Concerns about KRAS testing

Many physicians, when first asked about any concerns they might have regarding the test, reported that they perceived the test results to be straightforward, and that any ambiguous issues had already been dealt with by the test developers.

“I guess I don't really have any concerns per se … I believe the results that they come with, so if they [say] it's a wild-type then I believe that; if they say there's a mutation, I believe that” (28).

Similarly, many stated that they had no concerns, that the test “has been well standardized across all laboratories” (35); that they “trusted their pathology department to send it to a reliable laboratory” (38); that laboratories nowadays “are able to perform quite well” (61); or that “it's pretty mature technology to test for this gene mutation” (13).

However, as the interview progressed, several physicians began to express some concerns when asked specific questions about the test. Table 2 highlights the main concerns expressed along with a quote illustrating each. Most concerns were related to test validity and reliability and the overall quality of test results as well as the potential for false negatives and positives. Several respondents also noted that they felt uncomfortable interpreting data on the reliability and validity of the test and were unclear how to communicate these issues to their patients.

**Table 2:** Physicians’ perceived concerns with the KRAS test

Concerns with KRAS testing	Example/quote
Reliability and validity	“I always wonder about these fine-tuned testing. Because it is a make or break with treatment regimens for patients – how reliable the results are. When we submit a test to a lab for them to do it, we assume that we can trust them. Of course, that's not always 100 percent. There's always a false positive, false negative rate, right” (47).
Physician ability to interpret test results	“Honestly, I'm a clinician … I don't have the understanding to tell you, you know, if I have a concern or not” (60).
Test outcomes due to use of proper tissue specimen	“I don't know if you get the most benefit if it's the primary lesion or if it's a secondary lesion. I don't know if you test if the sample has – if it's a fresh sample or if you can get a block and test it many years later” (14).
Test does not predict treatment response	“The only thing is that you would rather have a test that tells you which patient responds, not which one doesn't respond. So it's kind of an – other, you know, a different way of looking at things. You know, we're used to, let's say, trying to get a test and say, Yes, you know – this is a tumor that is sensitive to this kind of treatment. Like you get HER2 and, you know, now – we can use Herceptin” (42).
Waste of resources	“…we can be harmed as a society if we just test people that we are not going [to give] the information [to], we're wasting our patients money, or members’ money and we can use something else, so it's really not reasonable to order it if there's no action that can be take[n] after the KRAS is done. So I find it as harm but not direct physical harm to the patient. Indirect harmto all of us, because that money is not used for something else. That's a waste of resources” (11).

Several respondents discussed their uncertainty over which biopsy sample to use when conducting the test, for example, whether to test old versus fresh biopsy tissue, or tissue from primary versus metastatic lesions. A smaller number of respondents expressed concerns with the value of the test itself. Additionally, some physicians voiced dissatisfaction that the test only predicts lack of response for those with a mutation, rather than success of response for those who are wild type.

### Oncologist–patient communication and decision making

We asked physicians about the nature of oncologist–patient communication and decision making related to the KRAS test and subsequent treatment.

#### Timing of KRAS communication

Most oncologists stated that they explain to their patients in advance that they will order a KRAS test, as a part of the long-term treatment planning process. To varying degrees, that explanation included spelling out the goals of therapy and what the patient could anticipate. However, many did not disclose in advance that they were ordering the test, and many discussed the test only when the patient had “progressed” and anti-EGFR treatment was under consideration. One physician noted the competing issues, “… if there is a very long conversation about the other aspects of their cancer care … I might just go ahead and order the test and talk to them about the results later on” (51). If the patient had “good performance status” there may be no immediate need for the discussion as the test results might be relevant at a later stage. Furthermore, some physicians saw no need to tell patients in advance because they considered the test to be standard of care.

### Role of guidelines

The national clinical guidelines appeared to serve several purposes for the oncologists. Guidelines provided a reference to ensure that oncologists were not missing an important issue, served as a handy summary enabling quick access, and was used as a useful reference point in conversations with patients. The main drawbacks of guidelines included the lag time, the breadth which may make them unsuitable for individual patient circumstances, and the fact that they could carry insurance enforcement ramifications.

Asked if it would be possible to prescribe anti-EGFR drugs to a patient without a KRAS test result, most responded that it would be possible, but unlikely that anyone would do so. In the words of one physician: “This isn't a test that somebody can order just willy-nilly. In our institution, this is going to be ordered pretty much exclusively by the medical oncologist – and there is no ten commandments about it or anything” (88). Most thought that there were no formal restrictions, although a few mentioned informal restrictions such as “reminders” or possible reimbursement issues. One oncologist described a computerized chemotherapy ordering system with automatic physician reminders being rolled out in his HMO.

## Discussion

While oncologists expressed a range of opinions about the KRAS test, there was a general consensus that the KRAS test was useful and provided benefits to mCRC patients. The KRAS test appeared to have been widely adopted in this population and oncologists in our sample were extremely positive about the test. This adoption pattern lends further explanation to another recent CERGEN study that found that almost 90% of oncologists across three different sites had ordered at least one KRAS test [12]. Also similar to the study by Webster and colleagues, oncologists in our study explained that they were less likely to test patients whose health and previous treatment response may limit the efficacy of anti-EGFR treatment.

While adoption was fairly widespread, oncologists administered the test at different time points within the patient's diagnosis and treatment and there was some confusion about which tumor sample to test (primary or metastatic lesions). Oncologists provided different rationales for testing at different time points. Rationales for testing at time of diagnosis of advanced CRC (stage III or IV) often focused on logistical issues around the need to test at the time the tumor biopsy was taken. On the other hand, several oncologists discussed the fact that some patients may not require anti-EGFR treatment (as they may die prior to needing second-line treatment) so they'd prefer to test when and if the patient began to fail first-line treatment. This is an important finding as the ASCO and NCCN guidelines are inconsistent about exactly when to test patients [9, 17]. Furthermore, cost-effectiveness studies have found the test to be cost-effective if administered at the time first-line treatment is failing [16]. In contrast, Webster and colleagues found that the time interval between mCRC diagnosis and testing has been declining [12]. It is important that future clinical guidelines provide clear guidance on tumor type to be tested and test timing and ensure that all variables (i.e., cost, efficiency, evidence, logistics, and physician preferences) are accounted for in this decision.

Finally, oncologists expressed support for the KRAS test and focused on the potential benefits of the test for their individual patients. However, when specifically queried, they also identified concerns with testing, related mainly to reliability and validity. Oncologists have voiced similar concerns with other tests that use genomic tumor profiling technologies such as those currently used for early-stage breast cancer [26, 27].

This study has several limitations. While the qualitative nature of this study provided a more in-depth understanding of how oncologists perceive KRAS testing, findings may not be indicative of physicians’ actual clinical practice patterns. Nevertheless, our findings were consistent with findings from Webster and colleagues which analyzed actual utilization patterns from electronic medical record data [12]. Although physicians in our study came from a number of different geographic locations, their clinical practices were mainly situated within a medium-large size integrated healthcare delivery system. Physicians interviewed were all affiliated with organizations that were part of the HMO Cancer Research Network. Thus, our findings may not be generalizable to other practice settings such as academic health centers or comprehensive cancer centers. Future research should explore the use of KRAS in a variety of different oncology practices. As this study relied on physician self-report, findings may be subject to social desirability bias and responder bias, and physicians may have overemphasized their usage or the importance they placed on the test. We attempted to mitigate this bias by asking the questions in a neutral manner, asking the questions in multiple different ways throughout the interview, and explaining that their answers were anonymous. Finally, these interviews were brief due to physicians’ time constraints therefore the data are somewhat constrained in terms of the breadth of topics about the KRAS test or about specific clinical encounters.

This study provides the only qualitative information to date examining how oncologists understand, discuss, and use a molecular diagnostic test in clinical practice. While KRAS is one test applied in a specific context of treatment decision making, the experience of KRAS testing is likely to be indicative of other molecular diagnostic tests used in oncology practice [28–30]. The rapid adoption and enthusiasm for KRAS suggests that these tests are likely filling an important informational need for oncologists when making treatment decisions. Future research should focus on the informational needs of patients around this test and whether patients feel informed or confident with their physicians’ use of these tests to determine treatment access.

Additional research is needed to account for the utilization patterns over time of these tests and how they influence treatment patterns in diverse clinical practices. While the rich qualitative data collected in our study may not be generalizable to all physicians and patients, the data highlight important factors that may be influencing KRAS adoption and use of KRAS testing in clinical decision making. Our results can be used to develop survey instruments in order to conduct follow-up studies that systematically survey oncologists in multiple practice settings to determine the impact of these different factors on KRAS adoption. This investigation into how oncologists adopted the test, perceived the test's benefits and limitations, and have used the test in clinical decision making provides an important context for operationalizing current clinical guidelines and developing guidelines for other molecular diagnostic tests in oncology.

## References

[1] Van Cutsem E, Kohne CH, Hitre E, Zaluski J, Chang Chien CR, Makhson A (2009). Cetuximab and chemotherapy as initial treatment for metastatic colorectal cancer. N. Engl. J. Med.

[2] Amado R, Wolf M, Peeters M, Siena E, Van Custem S, Freeman D (2008). Wild-type KRAS is required for panitumumab efficacy in patients with metastatic colorectal cancer. J. Clin. Oncol.

[3] Bokemeyer C, Bondarenko I, Makhson A, Hartmann JT, Aparicio J, de Braud F (2009). Fluorouracil, leucovorin, and oxaliplatin with and without cetuximab in the first-line treatment of metastatic colorectal cancer. J. Clin. Oncol.

[4] Hecht J, Mitchell E, Neubauer M, Burris H, Swanson P, Lopez T (2010). Lack of correlation between epidermal growth factor receptor status and response to panitumumab monotherapy in metastatic colorectal cancer. Clin. Cancer Res.

[5] De Roock W, Jonker DJ, Sartore-Bianchi F, Di Nicolantonio A, Tu D, Siena S (2010). Association of KRAS p.G13D mutation with outcome in patients with chemotherapy-refractory metastatic colorectal cancer treated with cetuximab. JAMA.

[6] McLellan E, Owen R, Stepniewska K, Sheffield J, Lemoine N (1993). High frequency of K-ras mutations in sporadic colorectal adenomas. Gut.

[7] Arber N, Shapira I, Ratan J, Stern B, Hibshoosh H, Moshkowitz M (2000). Activation of c-K-ras mutations in human gastrointestinal tumors. Gastroenterology.

[8] National Comprehensive Cancer Network (2010). http://www.nccn.org.

[9] Allegra CJ, Jessup JM, Somerfield MR, Hamilton SR, Hammond EH, Hayes DF (2009). American Society of Clinical Oncology provisional clinical opinion: testing for KRAS gene mutations in patients with metastatic colorectal carcinoma to predict response to anti-epidermal growth factor receptor monoclonal antibody therapy. J. Clin. Oncol.

[10] Blanke CD, Goldberg R, Grothey A, Mooney M, Roach N, Saltz L (2011). KRAS and colorectal cancer: ethical and pragmatic issues in effecting real-time change in oncology clinical trials and practice. Oncologist.

[11] Ciardiello F, Tejpar S, Normanno N, Mercadente D, Teague T, Wohlschlegel B (2011). Uptake of KRAS mutation testing in patients with metastatic colorectal cancer in Europe, Latin America, and Asia. Target. Oncol.

[12] Webster J, Kauffman T, Feigelson H, Pawloski P, Onitilo AA, Potosky AL (2013). KRAS testing and epidermal growth factor receptor inhibitor treatment for colorectal cancer in community settings. Cancer Epidemiol. Biomarkers Prev.

[13] Leighl N, Shepherd H, Butow P, Clarke S, McJannett M, Beale P (2011). Supporting treatment decision making in advanced cancer: a randomized trial of a decision aid for patients with advanced colorectal cancer considering chemotherapy. J. Clin. Oncol.

[14] Issa A, Hutchinson J, Tufail W, Fletcher E, Ajike R, Tenorio J (2011). Provision of personalized genomic diagnostic technologies for breast and colorectal cancer: an analysis of patient needs, expectations and priorities. Future Med.

[15] Mancl E, Kolesar J, Vermuelen L (2009). Clinical and economic value of screening for KRAS mutations as predictors of response to epidermal growth factor receptor inhibitors. Am. J. Health Syst. Pharm.

[16] Vijayaraghavan A, Efrusy M, Goke B, Kirchner T, Santas C, Goldberg R (2012). Cost-effectiveness of KRAS testing in metastatic colorectal cancer patients in the United States and Germany. Int. J. Cancer.

[17] National Comprehensive Cancer Network (2011). http://www.nccn.org.

[18] Wagner E, Greene S, Hart G, Field T, Fletcher S, Geiger A (2005). Building a research consortium of large health systems: the Cancer Research Network. J. Natl. Cancer Inst. Monogr.

[19] Scientific Software Development GmbH (2011).

[20] Glaser BG, Strauss AL (1967). The discovery of grounded theory: strategies for qualitative research.

[21] Strauss AL, Corbin JM (1998). Basics of qualitative research: techniques and procedures for developing grounded theory.

[22] Strauss AL, Corbin JM (1997). Grounded theory in practice.

[23] Denzin NK, Lincoln YS (2000). The discipline and practice of qualitative research. Handb. Qual. Res.

[24] Van Cutsem E, Nowacki M, Lang I, Cascinu S, Shchepotin I, Maurel J (2007). Randomized phase III study of irinotecan and 5-FU/FA with or without cetuximab in the first-line treatment of patients with metastatic colorectal cancer (mCRC): the CRYSTAL trial. J. Clin. Oncol.

[25] Jimeno A, Messersmith WA, Hirsch FR, Franklin WA, Eckhardt SG (2009). KRAS mutations and sensitivity to epidermal growth factor receptor inhibitors in colorectal cancer: practical application of patient selection. J. Clin. Oncol.

[26] Gradishar W, Hansen N, Susnik B (2009). Clinical roundtable monograph: a multidisciplinary approach to the use of oncotype DX in clinical practice. Clin. Adv. Hematol. Oncol.

[27] Mamounas E, Budd G, Miller K (2008). Incorporating the oncotype DX breast cancer assay into community practice: an expert Q&A and case study sampling. Clin. Adv. Hematol. Oncol.

[28] Haas J, Phillips KA, Liang S-Y, Hassett M, Keohane C, Elkin E (2011). Genomic testing and therapies for breast cancer in clinical practice. J. Oncol. Pract.

[29] Phillips KA, Liang S-Y, Van Bebber SL, Canpers Research Group (2008). Challenges to the translation of genomic information into clinical practice and health policy: utilization, preferences, and economic value. Curr. Opin. Mol. Ther.

[30] Linardou H, Briasoulis E, Dahabreh IJ, Mountzios G, Papadimitriou C, Papadopoulos S (2011). All about KRAS for clinical oncology practice: gene profile, clinical implications and laboratory recommendations for somatic mutational testing in colorectal cancer. Cancer Treat. Rev.

